# Single-molecule modeling of mRNA degradation by miRNA: Lessons from data

**DOI:** 10.1186/1752-0509-9-S3-S2

**Published:** 2015-06-01

**Authors:** Celine Sin, Davide Chiarugi, Angelo Valleriani

**Affiliations:** 1 Department of Theory and Bio-Systems, Max Planck Institute of Colloids and Interfaces, Science Park Golm, Potsdam, Germany

**Keywords:** miRNA, mRNA complex degradation, mRNA decay, stochastic modeling, single molecule

## Abstract

Recent experimental results on the effect of miRNA on the decay of its target mRNA have been analyzed against a previously hypothesized single molecule degradation pathway. According to that hypothesis, the silencing complex (miRISC) first interacts with its target mRNA and then recruits the protein complexes associated with NOT1 and PAN3 to trigger deadenylation (and subsequent degradation) of the target mRNA. Our analysis of the experimental decay patterns allowed us to refine the structure of the degradation pathways at the single molecule level. Surprisingly, we found that if the previously hypothesized network was correct, only about 7% of the target mRNA would be regulated by the miRNA mechanism, which is inconsistent with the available knowledge. Based on systematic data analysis, we propose the alternative hypothesis that NOT1 interacts with miRISC before binding to the target mRNA. Moreover, we show that when miRISC binds alone to the target mRNA, the mRNA is degraded more slowly, probably through a deadenylation-independent pathway. The new biochemical pathway proposed here both fits the data and paves the way for new experimental work to identify new interactions.

## Introduction

### Background

In living cells, the level of protein expression is thoroughly regulated. Many crucial processes for this regulation occur at the post-transcriptional level. In this context, control mechanisms acting on messenger RNAs (mRNAs) play a pivotal role. A number of biochemical pathways converging on cytosolic mRNAs serve to enhance or repress gene expression. These pathways are known to operate by enhancement of translation [1,2], repression of translation [3,4] or modulation of mRNA lifetimes [3-6]. The global picture emerging from the growing body of experimental evidence depicts a complex interaction network which affects the mRNAs available for translation. This network is composed of several biochemical pathways, often interwoven and cross-talking [7,8], involving mRNA binding proteins as well as non coding RNAs [9-11]. While there are a number of mechanisms responsible for mRNA degradation in eukaryotic cells [9], the decay of messages mediated by micro-RNAs (miRNAs) plays a prominent role in the control of gene expression [3,12,13].

Despite extensive study, the topology and dynamics of miRNA-mediated mRNA degradation pathway are still unclear. One of the main challenges stems from the fact that intermediate states of the pathway are unknown or difficult to quantify; experimentally, it is only feasible to measure the decay patterns of the target mRNAs. Bridging the gap between observed *decay patterns *and *degradation pathways *is non-trivial [14], since the former refer to a population average and the latter refers to the single-molecule stochastic process of degradation. Here we apply a rigorous strategy to reconstruct the miRNA-mediated degradation pathway, starting from experimentally measured decay patterns. Surprisingly, we find the previously proposed pathway not consistent with the experimental data. We propose an alternative model which fits the decay pattern and allows us to gain some insight into the network topology.

### The experimental data

**Figure 1:**
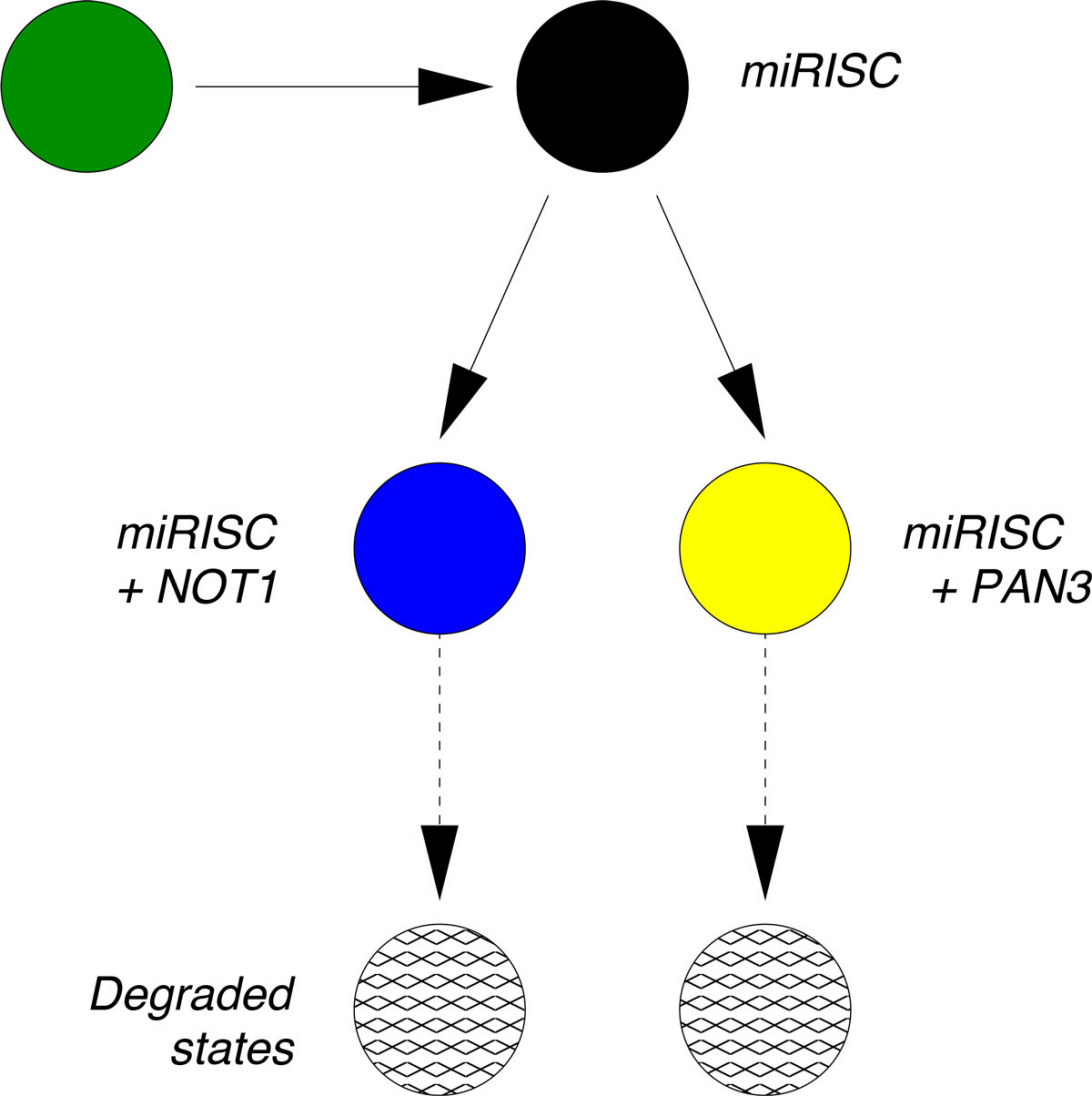
**The biochemical degradation network hypothesized by Braun *et al***. [15] for the degradation of a target mRNA by miRNA in *D. melanogaster *S2 cells. According to this network, the mRNA in its initial state (green circle) first binds to the miRISC complex thus leading to a new biochemical state (black circle). The miRISC and its target then recruit the proteins NOT1 and/or PAN3, leading to the two states indicated with the blue and yellow circle, respectively. From these two states the mRNA is finally degraded through a complex sequence of events including deadenylation followed by decapping [3]. This unspecified sequence of events is indicated with dotted arrows in all figures of this paper.

**Figure 2:**
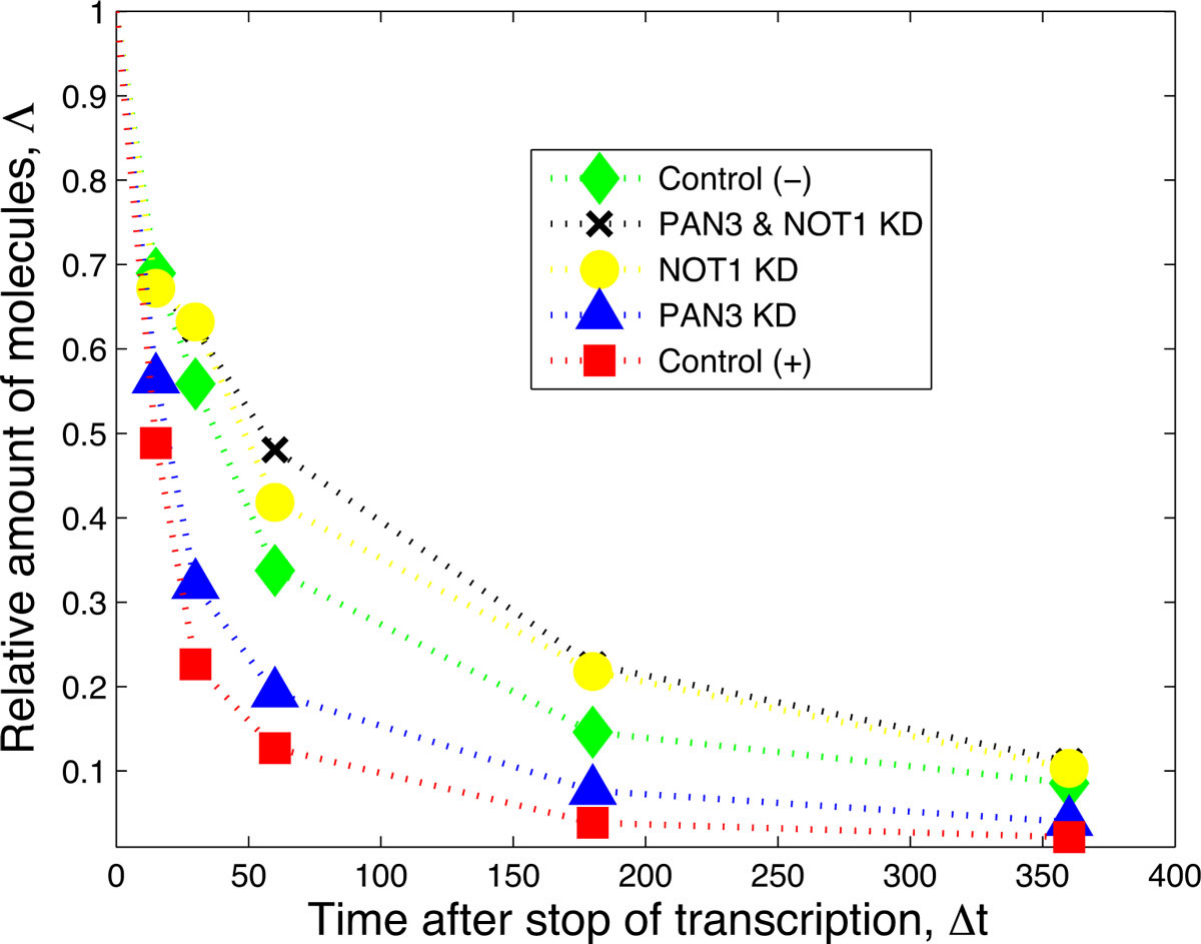
**The experiment performed by Braun *et al***. [15] consists of knocking down several permutations of the target's degradation factors. The "Control (-)" data result from an experimental setup in which the miR9-b is not expressed; the "PAN3 & NOT1 KD" data result from an experimental setup in which only the miR9-b is expressed but both NOT1 and PAN3 are knocked down; the "NOT1 KD" data result from a setup in which miR9-b and PAN3 are expressed while NOT1 is knocked down; the "PAN3 KD" data result from a setup in which miR9-b and NOT1 are expressed while PAN3 is knocked-down; the "Control (+)" data result from a setup in which all three factors are expressed. The data have been extracted from figure 7A of [15]. The numerical values are reported in the Additional file 1.

To clarify the interplay of the various factors in a degradation pathway involving miRNA, the protein complexes NOT1, and the protein complex PAN3, Braun *et al*. [15] performed a series of controlled knockdown experiments in *D. melanogaster *S2 cells containing constructs for the miRNA miR-9b, its target mRNAs F-Luc-Nerfin mRNAs, and the factors NOT1 and PAN3, which are known to trigger mRNA deadenylation. In each experiment, a subset of NOT1, PAN3 and/or miR-9b were selectively knocked down, yielding cell lines expressing different combinations of those factors: a control line without the miR-9b, a cell line with miR-9b only, a cell line with NOT1+miR-9b (but not PAN3), a cell line with PAN3+miR-9b (but not NOT1), and finally a cell line with all three factors NOT1+PAN3+miR9-b. A graphical representation of the network of biochemical interactions hypothesized in [15] is presented schematically in Figure 1. After steady state expression of the factors, the transcription of mRNA was blocked and the decay patterns over time for three independent biological replicas were measured. The average decay patterns from these three replicas were reported in [15]; we extract these patterns and present them in Figure 2.

The conclusion of this detailed experimental study is that NOT1 is a more relevant factor than PAN3 in destabilizing the mRNA [15]. When only NOT1 is knocked down, the decay of F-Luc-Nerfin mRNA is significantly slower (yellow curve, Figure 2) than the control (red curve, Figure 2). In contrast, the effect of PAN3 knock down is less significant (blue curve, Figure 2). These findings apparently confirm that the degradation pathway through NOT1 in Figure 1 is the most prominent pathway for degradation of the target mRNA. Although this conclusion is relatively robust, the published analyses do not validate the hypothesized biochemical degradation pathway given in Figure 1. Indeed, in the negative control (green curve in Figure 2), the miRNA is knocked-down so that the formation of a specific silencing complex miRISC is suppressed, yet the target mRNA still decays. Additionally, when only the miRNA is expressed while PAN3 and NOT1 are knocked-down, the target mRNA decays (black curve in Figure 2), but is definitively more stable than in the negative control. Both of these cases suggest that the model hypothesized in Figure 1 should be expanded to include additional degradation pathways.

An important conceptual consideration is that Figure 1 depicts degradation from the single-molecule perspective whereas the curves in Figure 2 are averages as a function of time. Therefore, our strategy consists of starting with the network shown in Figure 1 and validating it against the experimental decay patterns. At the same time, we will propose alternative parsimonious extensions of the network when the validation fails. In particular, we will find that when the miRISC complex interacts with the mRNA alone, it seems to stabilize the mRNA and perhaps trigger a deadenylation independent degradation of the mRNA. Furthermore, we will show that the data supports the hypothesis that miRISC binds to NOT1 *before *recruiting the target mRNA and that there is a strong enhancement of mRNA recruitment when PAN3 is also present.

## Methods

As previously mentioned, the relationship between *degradation pathways *(such as the one in Figure 1) and *decay patterns *(such as those in Figure 2) is not trivial. If the decay pattern was exponential, the halftime of the mRNA population estimated from the decay pattern would be directly related to the rate of decay of single molecules. The analysis of the decays shown in Figure 2 shows that a model based on a single exponential results in a poor fit; more complex models are preferred even in light of evaluations based on the Akaike Information Criteria (AIC). Furthermore, if the decay curves could be well described by a model based on a single exponential, the traces would appear as straight lines when plotted in a linear-log scale (see Figure 7 in [15]).

**Figure 3:**
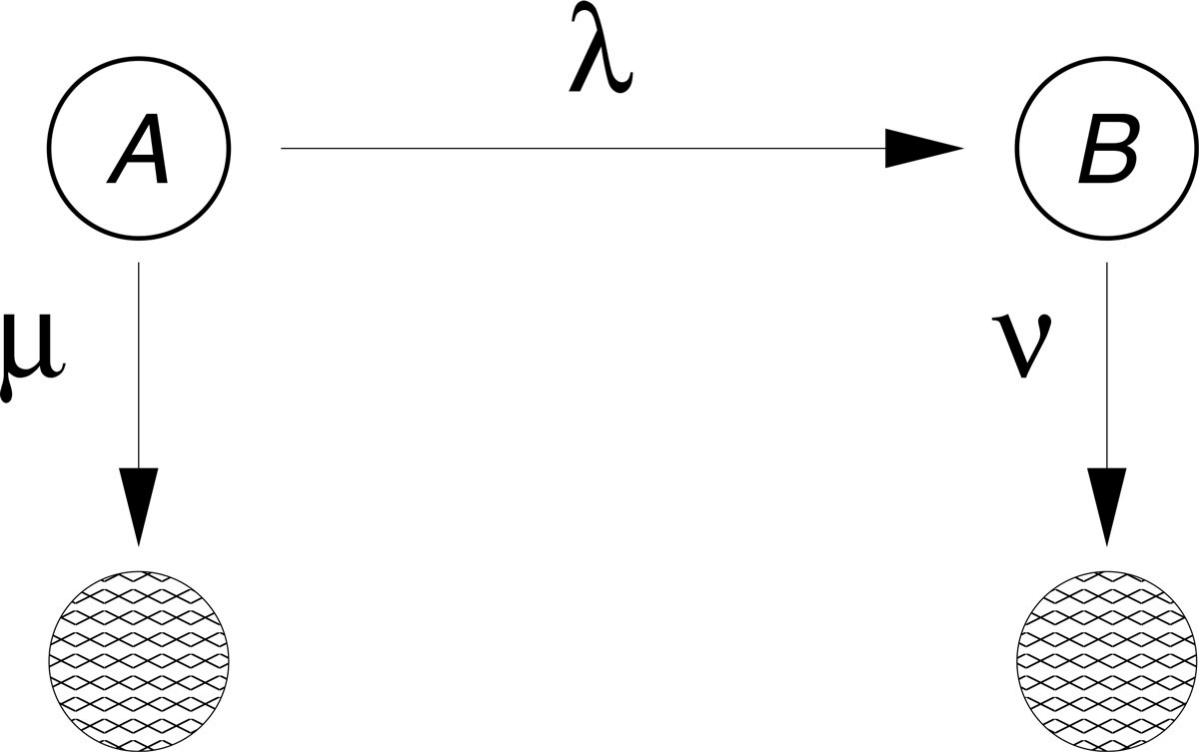
**Parsimonious two-state model. **This two-state model is able to fit all of the decay patterns in Figure 2, by an appropriate choice of the three fitting parameters, λ, µ, ν. In this context, the mRNA is initially in the state A. From this state it can be degraded directly with a rate µ or change biochemical state, with rate λ, from which it is then degraded with rate ν. Although this network performs a better fit of the data and is preferred to the exponential fitting based on the AIC criterion, the interpretation of the states is unclear. The fitting curves and the parameters are discussed in Additional file 1.

The issue of relating complex degradation pathways to decay patterns has been tackled in [14,16]. In [14] it was shown that decay patterns similar to those depicted in Figure 2 can be generated by single-molecule networks satisfying certain properties, if one assumes that the transitions between biochemical states can be modeled as first-order chemical reactions. The mathematics supporting this reasoning was presented in [14] and is summarized in the Additional file 1 where we show how to derive the necessary mathematical functions using first passage time methods [17-21]. In order to generate decay patterns such as those in Figure 2, the corresponding single-molecule degradation pathway must be composed of at least two states from which degradation is possible. Thus, in principle, one can either hypothesize a network of states that represents the biochemical pathway of degradation based on predictions and prior knowledge, or one can use the mathematical relationships mentioned above to find the most parsimonious network that fits that data. The most parsimonious network of states that is able to fit each of the curves in Figure 2 is the two-state model depicted in Figure 3.

While the network in Figure 3 results in a definitively better fit to the data and thus could be used to derive quantities such as the average lifetime and the age dependent degradation rate, it does not tell us if the network in Figure 1 is a suitable framework for the decay patterns observed in Figure 2. To address this question we employ a hierarchical strategy: (i) we start by fitting the negative control decay pattern (the green trace "Control (-)" in Figure 2) to the most parsimonious model (Figure 3) and fix the corresponding three rates; (ii) we then consider the next decay pattern with one additional decay factor active and enlarge the network of states to accommodate the additional decay factor. We continue until each curve has been evaluated and the corresponding network is built.

The details of the functions used to perform the fit can be found in Additional file 1: Section S1.1 provides the general aspects of the mathematical background required for the purpose of this paper, section S1.2 gives the explicit formulas used for the fit, section S1.4 provides parameters estimations.

## Results

Based on the hierarchical strategy above, we start with the "Control (-)" curve (green decay pattern, Figure 2). This curve describes the decay of the mRNA when none of the degradation factors (miRNA, NOT1 and PAN3) are present. In the framework of the hypothesized network in Figure 1, this corresponds to all downstream processes inactive, thus reducing the network to just the first state (green circle). This is obviously not sufficient to explain the observed decay, since a single state without decay would produce a horizontal line (*i.e*. no decay). This one-state scenario is also not consistent with biological reality. Indeed, even the most stable cellular macromolecule is eventually degraded. In fact, there are many biochemical pathways devoted to mRNA degradation [9].

**Figure 4:**
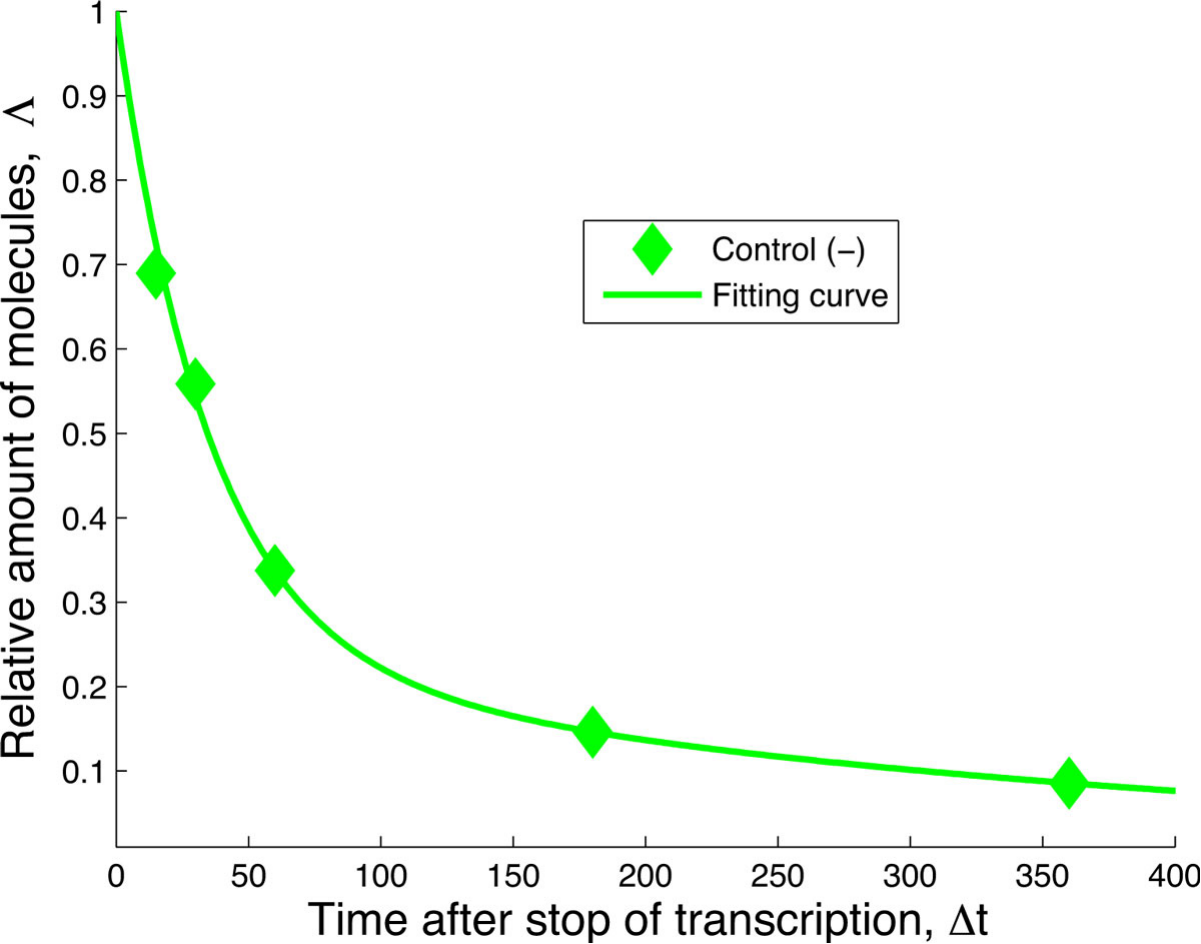
**Fit of the negative control data.** The negative control decay pattern can be fitted with the single molecule network from Figure 3. This delivers the rates λ, µ and ν that will be kept constant through any successive enlargement of the network when considering the other decay patterns. The values of the rates are: λ = 0.0008 min^−1^, µ = 0.0276 min^−1^, ν = 0.0028 min^−1^, with confidence intervals [0.0002, 0.0013], [0.0229, 0.0324], [0.0018, 0.0038], respectively.

**Figure 5:**
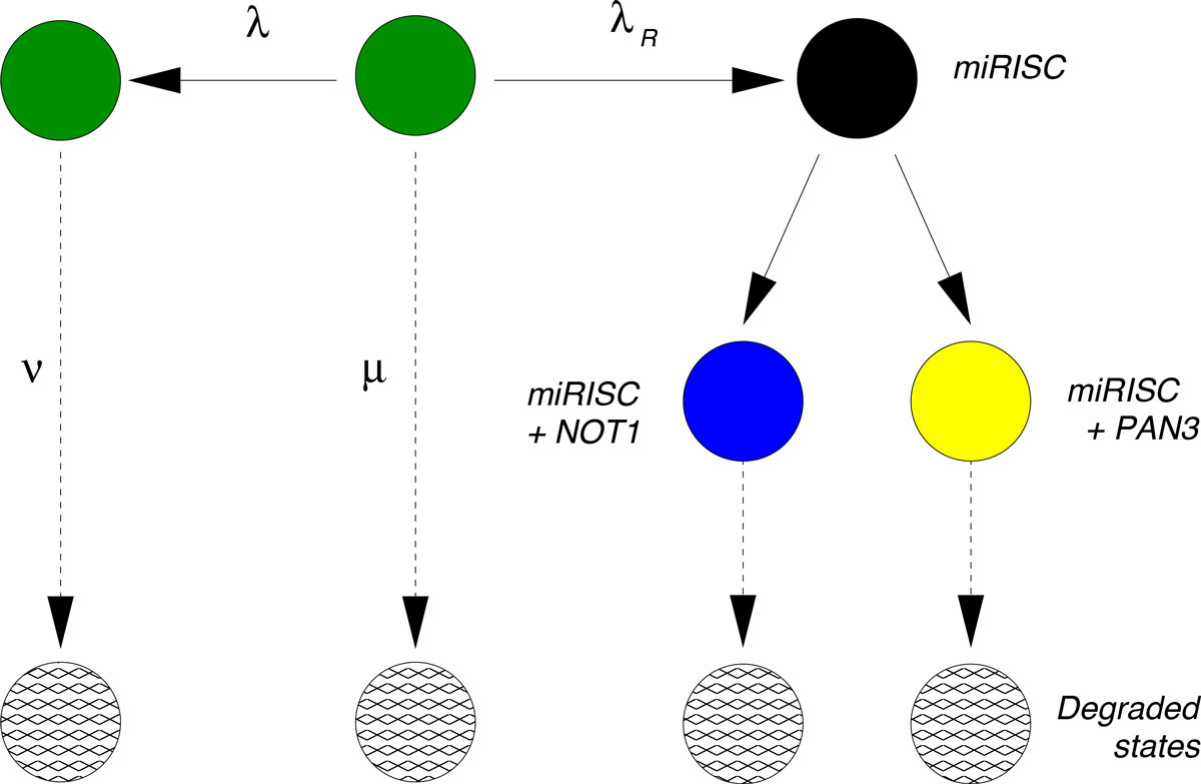
**The initial hypothesis of Braun et al**. [15] is complemented with an alternative pathway that competes with the miRNA-mediated pathway. Initially, all mRNA start at the state represented by the central green circle and follow one of the two competing paths. The path towards the left (denoted by λ) is exclusive of the miRISC pathway (towards the right, denoted by λ_R_ ), and vice versa. The rates λ, µ and ν have been fixed through the fitting performed in Figure 4. The negative control "Control (-)" data result from the decay pattern of the mRNA when only the states represented by the green circles are available.

In the absence of further information, we fit the green "Control (-)" curve of Figure 3 to the most parsimonious (or minimal) network that still captures the dynamics of the data (Figure 4). We can interpret the need for such network by saying that in absence of miRNA-dependent degradation, mRNA molecules can be degraded through two pathways, differing by their kinetic features (see Figure 3). The first class of pathways (governed by the rate *µ *in Figure 3) is characterized by a single step and it can be representative of the set of "constitutive" reactions which target mRNAs non-specifically and are catalyzed by enzyme complexes such as the exosome [9]. The second class of pathways (along the path *λ *and *ν *in Figure 3) exhibits two steps and represents the degradation processes (independent of miRNAs) passing through a control step of a more complex degradation pathway (e.g. the preliminary binding of specific proteins to the target mRNA). One such example independent of miRNA and the NOT1/PAN3 factors is the ARE-mediated degradation pathway [6,22]. We should stress that the network to fit the green "Control(-)" curve of Figure 3 was not foreseen in the pathway proposed in [15] and that we introduce it in order to consistently perform a fit for each of the experimental decay patterns. This gives rise to an enlarged network of biochemical interactions, shown in Figure 5. The existence and the strength of such additional degradation pathway may be dependent on the species of mRNA and on the growth conditions of the cell culture.

### The crisis of the original hypothesis

**Figure 6:**
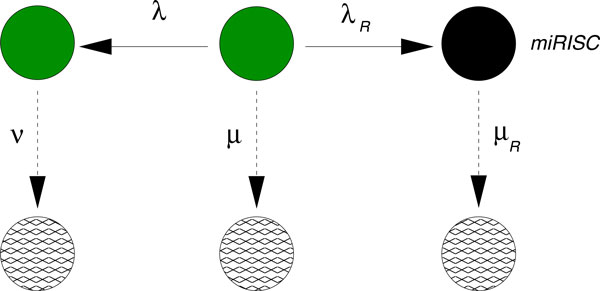
**Network to fit the data NOT1 & PAN3 KD.** After fixing the rates µ, λ and ν from the fitting of the negative decay pattern in Figure 4, we use this network in order to model the decay pattern when miRNA is expressed but NOT1 and PAN3 are knocked down. In order to perform this fit, however, we must add a new transition to degradation after the binding of the miRISC (rightmost transition to degradation). This new transition might contain a complex set of processes which are most likely independent of deadenylation. In the light of recent results, this arrow could include deadenylation-independent decapping [23,24].

At the next level of our hierarchical approach we consider the decay pattern that results when the miRNA is expressed but NOT1 and PAN3 are knocked-down (black decay pattern in Figure 2). When PAN3 and NOT1 are knocked down, the arrows from the black state to the blue and yellow states in Figure 5 are absent, resulting in the right pathway having no transition to degradation. However, if we look at the corresponding decay pattern (black line in Figure 2), we realize that such a structure is not compatible with the data because a vertex without transition to degradation would imply a flattening of the curve to a steady state amount of mRNA, corresponding to the amount of mRNA arrested in this rightmost state (black circle). To model the observed decay of mRNA, we need to postulate an additional transition to degradation from the state obtained after binding with miRISC, as shown in Figure 6. A possible interpretation is that the additional transition includes unknown biochemical degradation pathways which are independent of deadenylation. Supporting this hypothesis are the findings reported in [23]: they report that the binding of the miRISC complex to the target mRNA can promote the dissociation of Poly-A-Binding Proteins (PABPs). PABPs are known to protect the poly-A tail of the mRNA from being hydrolyzed, thus stabilizing the mRNA. Thus, miRNA-mediated PABP dissociation can trigger NOT1- and PAN3-independent deadenylation, which eventually leads to degradation of the mRNA [23].

**Figure 7:**
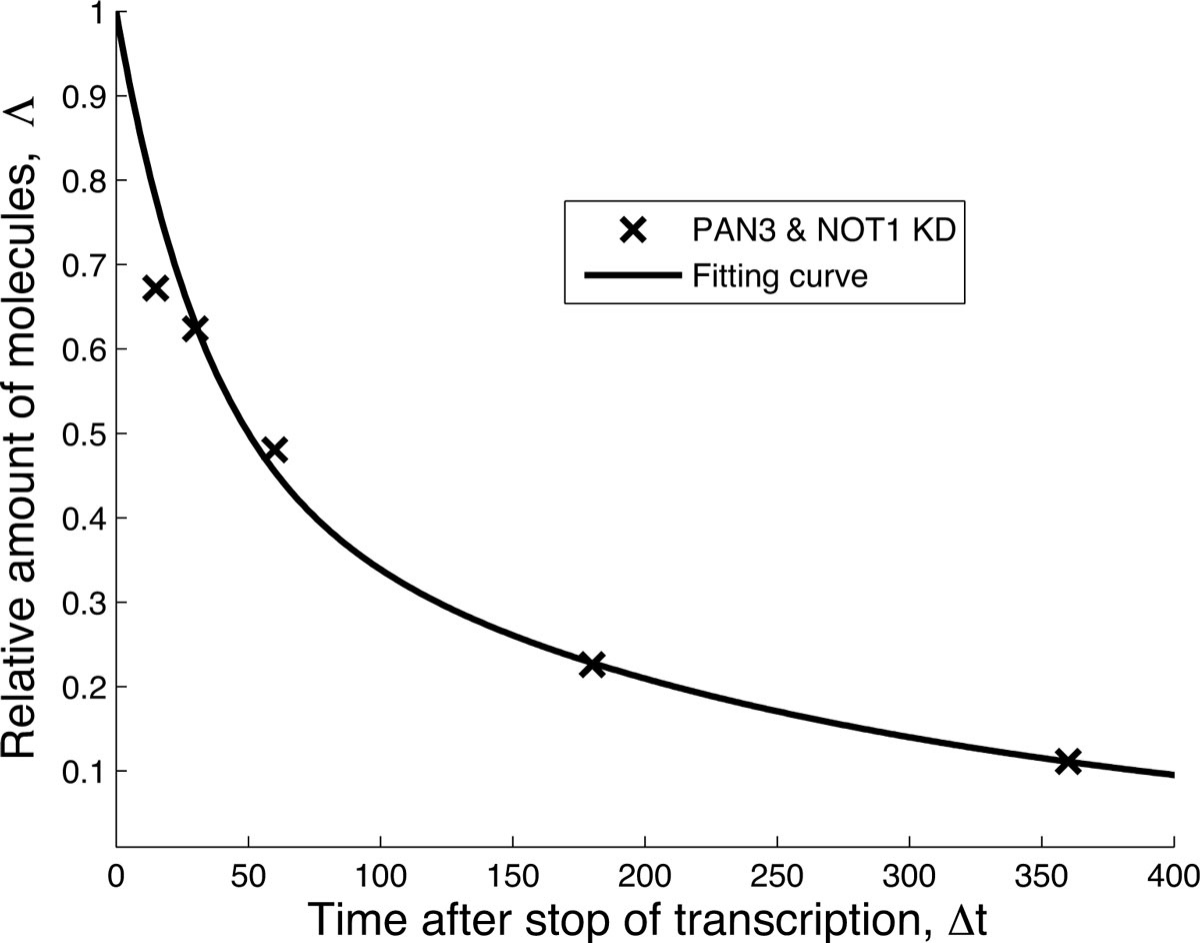
**Fit of the data NOT1 & PAN3 KD.** After fixing the rates µ, λ, ν from the fitting of the negative decay pattern in Figure 4, we use the network in Figure 6 to fit the decay pattern when miRNA is expressed but NOT1 and PAN3 are knocked down. The values of the rates are λ_R_ = 0.0023 min^−1^ and µ_R_ = 0.0052 min^−1^ with 95% confidence intervals [0, 0.0052] and [0.0030, 0.0074], respectively.

Fitting the data to the network depicted in Figure 6 reveals several crucial aspects of the hypothesized network of Figure 1 and shows the shortcomings of the latter. While the fit of the data using the network in Figure 6 works pretty well (see Figure 7), it fixes the rate *λ_R _*associated to the binding of miRISC on the mRNA. This rate is therefore independent of the transitions occurring downstream.

The next step in our hierarchical program, however, would be to take the next decay patterns and fit them to the network given in Figure 5 activating the appropriate pathway depending on which factor (NOT1 and/or PAN3) is present while keeping *λ_R_* fixed. Before doing that, however, a simple computation shows that the fraction of mRNA going through the miRISC pathway is given by

(1) $$\sigma_{R}=\frac{\lambda_{R}}{\lambda_{R}+\lambda +\mu }\sim 0.074,$$

*i.e*., about 7% of the whole mRNA binds to miRISC complexes in the absence of NOT1 and or PAN3, based on the network shown in Figure 6. This discovery leads to two conclusions. First, the fraction of mRNA that can be manipulated after binding with miRISC is so small that an enlargement of the network by including a separate NOT1 and a separate PAN3 pathway downstream of miRISC binding becomes meaningless. Indeed, attempts to do so lead to very poor fitting of the remaining curves (see Additional file 1). Second, such a small fraction of miRNA-regulated mRNA (about 7%) would indicate that miRNA cannot be considered a strong mechanism of gene regulation, contrary to the experimental evidence that miRNA is a strong regulatory mechanism.

Therefore, we are forced to partially reject the hypothesis formulated in Figure 1 and revise it in search for other possible interactions between miRISC, PAN3 and NOT1. Note that the computed value of 7% is necessarily affected by some error due to the precision by which the data could be extracted from the plots originally published in Ref. [15]. Nevertheless, this value is an indication that the model of degradation originally proposed in [15] would predict that only a very small fraction of mRNA is involved in miRNA mediated degradation. In the following we will present a parsimonious model of degradation that is able to predict more realistic figures of the relative amounts of mRNAs involved in the different degradation pathways.

Finally, the comparison between the decay pattern fitted in Figure 4 and 7 shows that binding of miRISC alone does stabilize the mRNA compared to when the miRNA is not expressed. This is a strong indication that miRISC "protects" the target mRNA from the action of alternative, competing degradation pathways.

### A new hypothesis arises from the data

**Figure 8:**
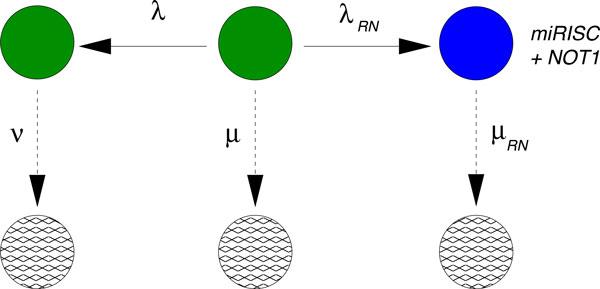
**Biochemical network fitting the data when PAN3 has been knocked down**. After fixing the rates µ, λ and ν from the fitting of the negative decay pattern in Figure 4, we use this network in order to model the decay pattern when miRNA is expressed but PAN3 is knocked down ("PAN3 KD" data). The transition from the central green state to the state with the mRNA bound to the preformed complex miRISC + NOT1 is ruled by the transition rate λ_RN_. The downwards transition, ruled by the rate µ_RN_ includes several steps that cannot be specified from these data.

**Figure 9:**
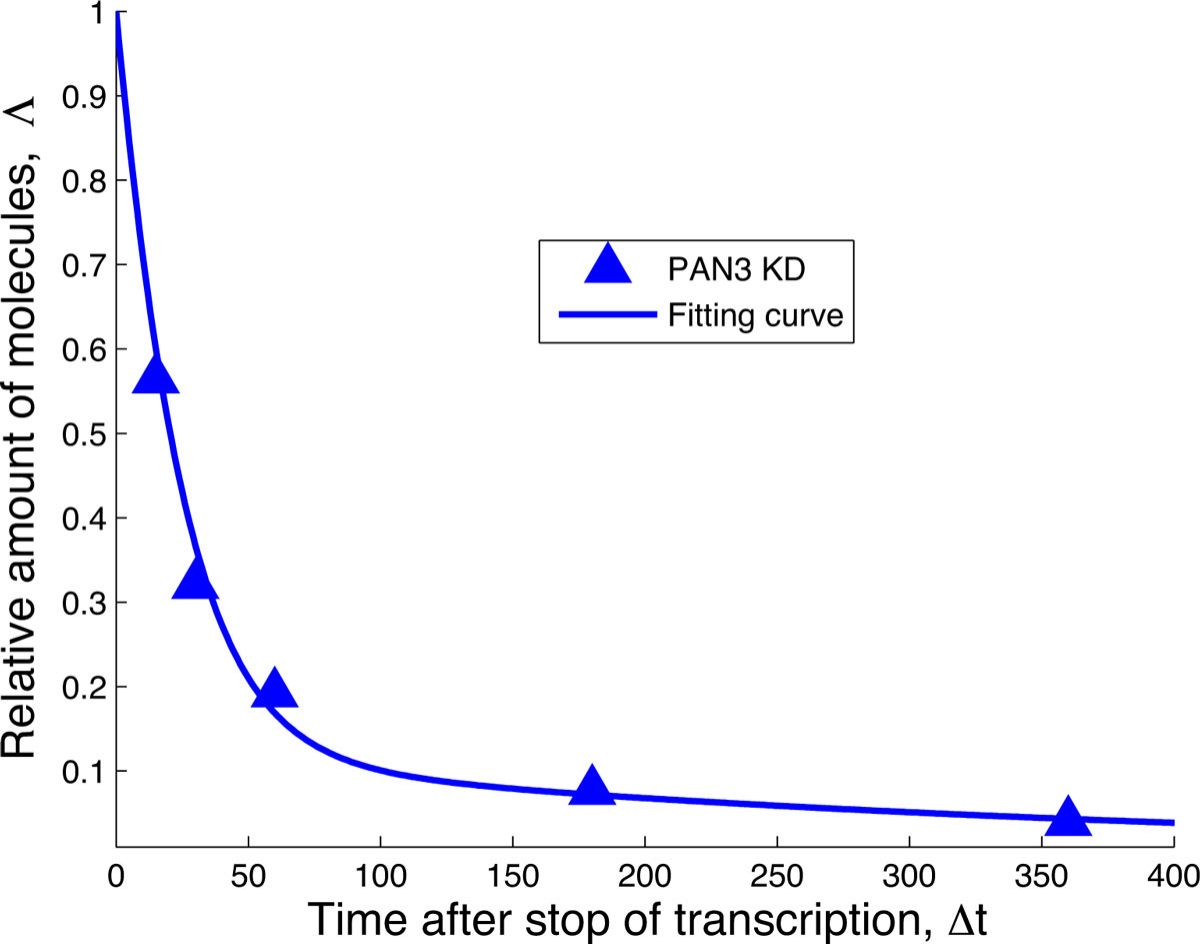
**Fit of the data PAN3 KD.** After fixing the rates µ, λ, ν from the fitting of the negative decay pattern in Figure 4, we use the network in Figure 8 in order to fit the decay pattern when miRNA is expressed but PAN3 is knocked down. The values of the rates are λ_RN_ = 0.046 min^−1^ and µ_RN_ = 0.0461 min^−1^ with 95% confidence intervals [0.0305, 0.0687] and [0.0319, 0.0602], respectively. These rates indicate that in the absence of PAN3, about 64 % of the mRNA in the experiment are degraded by the action of miRISC.

Since the initial hypothesis that miRISC binds to the mRNA and then recruits the NOT1 molecule does not result in a reasonable fit, we can hypothesize that miRISC binds to NOT1 *before *recruiting the target mRNA. This hypothesis is formulated in Figure 8, which can be used to fit the data where only the PAN3 complex has been knocked down. The fit is indeed very good, as seen in Figure 9. Based on this result, the sole effect of NOT1 binding to miRISC leads to a strong increase in the percentage of mRNA degraded through miRISC activity, given by

(2) $$\sigma_{RN}=\frac{\lambda_{RN}}{\lambda_{RN}+\lambda +\mu }\sim 0.64,$$

which emphasizes the strong role of NOT1 in the degradation of mRNA.

**Figure 10:**
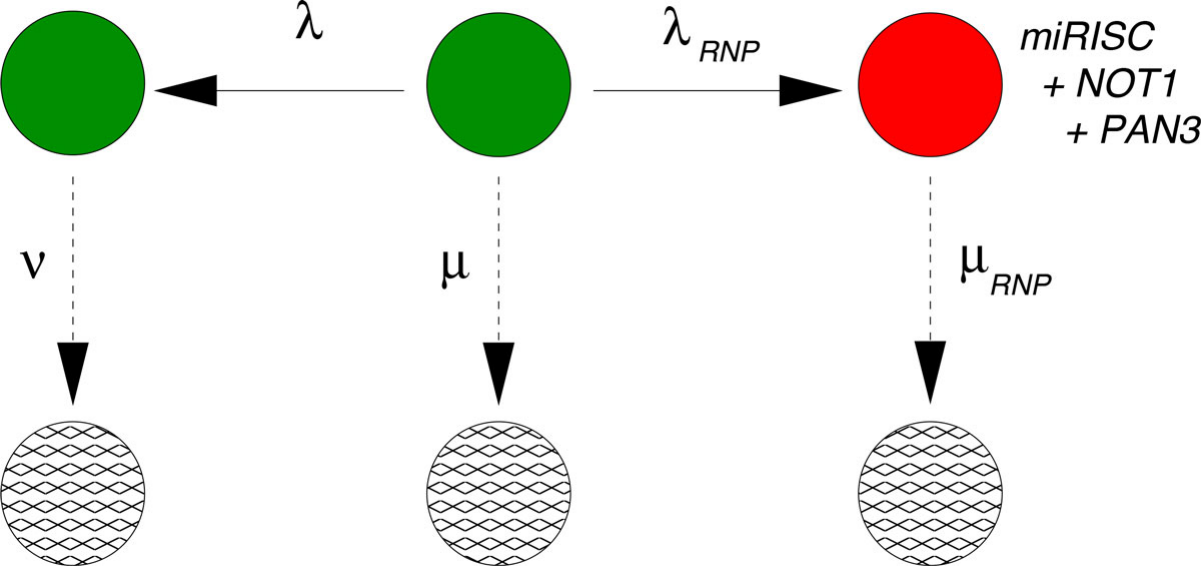
**Network of the "Control(+)" data.** Biochemical network able to fit the data when all three factors miRISC, NOT1 and PAN3 are expressed. After fixing the rates µ, λ and ν from the fitting of the negative decay pattern in Figure 4, we use this network in order to model the decay pattern when all three factors are expressed. The transition from the central state (green) to the state with the mRNA bound to the preformed complex miRISC + NOT1 + PAN3 is ruled by the transition rate λ_RNP_. The downwards transition, ruled by the rate µ_RNP_ includes several steps that cannot be specified from these data.

**Figure 11:**
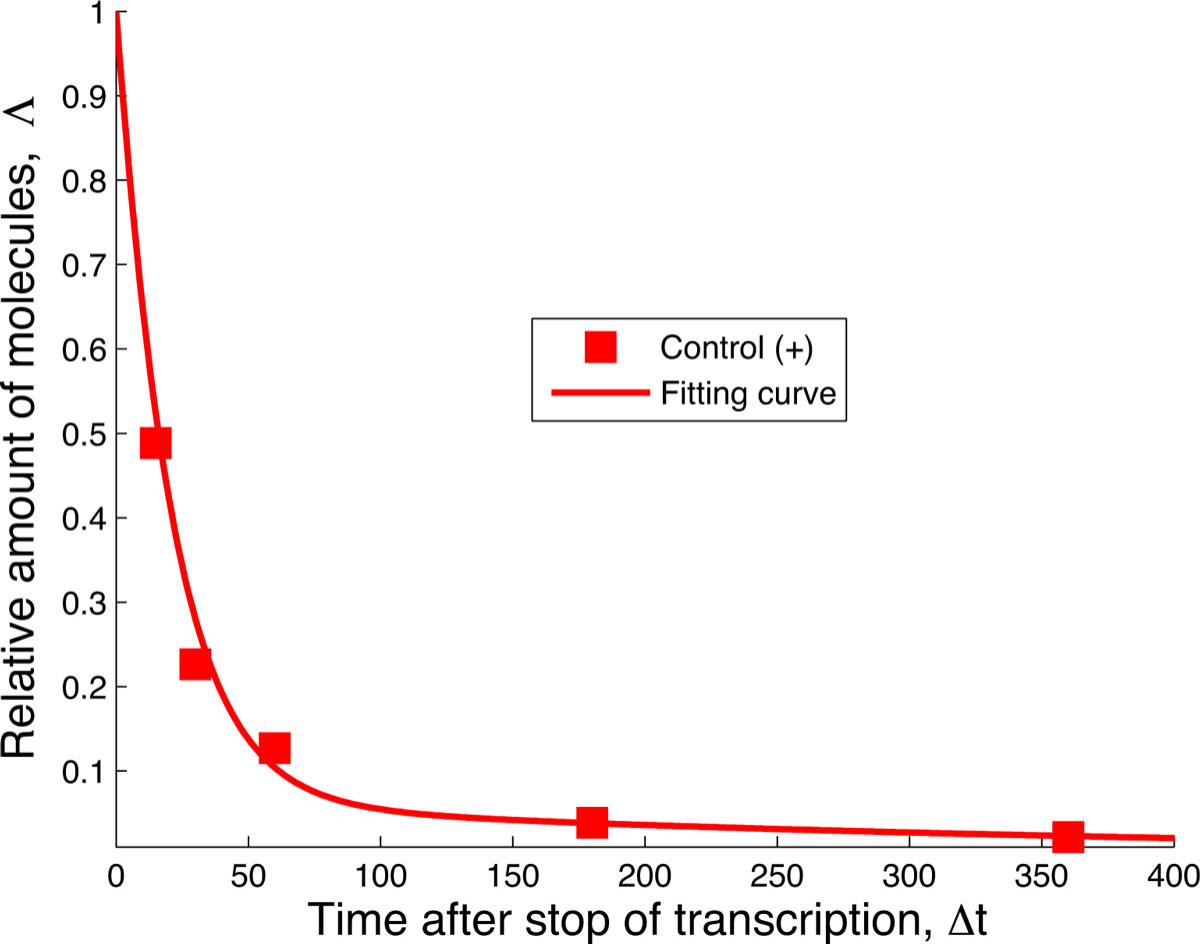
**Fit of the "Control(+)" data.** After fixing the rates *µ, λ, ν *from the fitting of the negative decay pattern in Figure 4, we use the network in Figure 10 in order to fit the decay pattern when all three factors, miRNA, NOT1 and PAN3 are expressed. The values of the rates are *λ_RNP _*= 0.1501 ^−1^ and *µ_RNP _*= 0.0493 min^−1^ with 95% confidence intervals [0.0908, 0.2094] and [0.0373, 0.0612], respectively. These rates indicate that when both NOT1 and PAN3 are expressed together with the miRNA, about 84% of the mRNA in the experiment are degraded by the action of miRISC.

The final curve of the experiment in [15] concerns the action of all the factors together. Based on the results obtained so far in Figures 8 and 9 there may be several hypotheses about the possible combined action of PAN3 and NOT1. Since PAN3 alone (yellow curve in the original data shown in Figure 2) does not have a significant effect on the decay of the mRNA compared to the action of miRISC alone, we conclude that PAN3 works cooperatively with NOT1 by forming a complex miRISC+NOT1+PAN3 before binding to the target mRNA. This hypothesis is formulated in Figure 10. The data fits the network in Figure 10 quite well, as one can see in Figure 11. By using the values *λ_RNP _*and *µ_RNP _*we can again compute the fraction of target mRNA that is degraded by the action of miRISC+NOT1+PAN3:

(3) $$\sigma_{RNP}=\frac{\lambda_{RNP}}{\lambda_{RNP}+\lambda +\mu }\sim 0.84,$$

indicating that this model produces the strong regulatory effect of the miRNA on its target as expected.

### The cooperative role of PAN3

**Figure 12:**
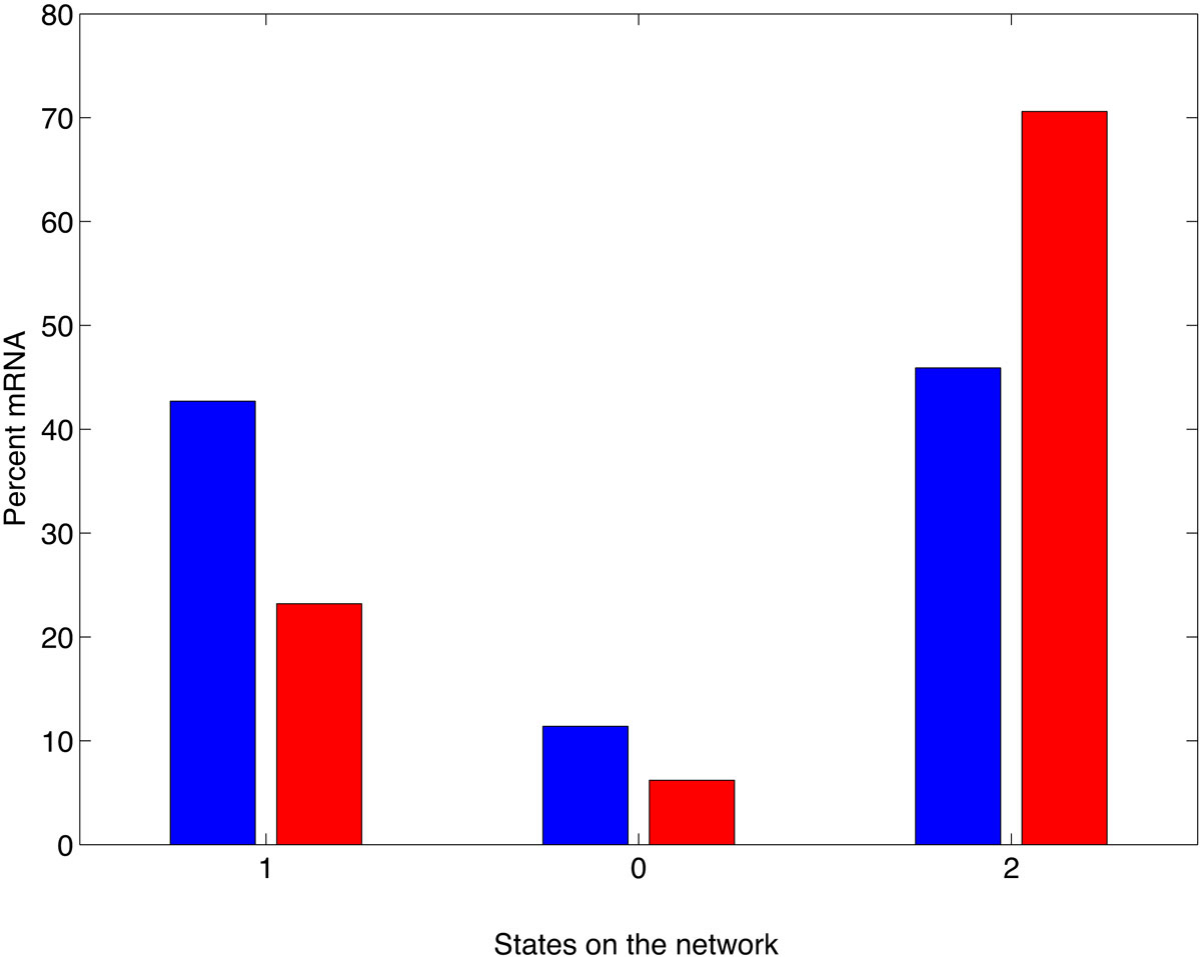
**Cooperative effect of PAN3.** Percent of mRNA at steady state expression level in each of the three main states of the networks in Figures 9 and 11. State 0 represents the mRNAs that are not bound to miRISC and not bound to the competing alternative complexes. The alternative degradation pathways lead to state 1 whereas the miRISC-mediated pathway leads to state 2. The blue histogram refers to the case when only the miRNA and NOT1 are expressed whereas the histogram in red refers to the experiments when all three factors miRNA, NOT1 and PAN3 are expressed. The percentage of mRNA in state 2 is strongly increased by the expression of PAN3 at the expenses of the amount of mRNA in state 1. Since the rates *µ_RNP _*and *µ_RN _*do not differ, this shift in the amount of mRNA between the states is due to a strong increase in the rate *λ_RNP _*compared to *λ_RN _*. This indicates that the most important role of PAN3 is to shift the balance towards miRNA-mediated decay.

We have seen that the expression of PAN3 in a system with miRNA and NOT1 strongly destabilizes the target mRNA and shortens its lifetime. Nevertheless, we can better understand the role played by PAN3 in cooperation with NOT1, when we compare the fraction of target mRNA that are expected to be found in the "miRISC+NOT1" state in Figure 9 (blue circle) with the fraction of target mRNA to be found in the "miRISC+NOT1+PAN3" state in Figure 11 (red circle). This comparison is made in Figure 12. There, we find the fraction of mRNAs in each of the three states after denoting state 0 the state in the middle of the network, state 1 the state on its left side and state 2 the state on the right side (blue circle in Figure 9 and red circle in Figure 11). We can see from the bar plot that the major contribution of PAN3 is to shift the balance in favor of the miRNA by subtracting target mRNAs to the alternative pathway. By expressing PAN3, the amount of mRNA that are found in state 1, corresponding to the mRNA bound to protein complexes competing with the miRISC, decreases by almost 20% of the total mRNA, whereas the amount found in state 0 decreases by only a 5% of the total. This indicates that the major role played by PAN3 is not to enhance deadenylation but rather to enhance the recruitment of the target mRNA at the expenses of alternative degradation pathways that do not involve miRNA. From the available data it is not possible to establish if the mRNA in these three states are also translational competent or silenced. From the biochemical point of view, each of these three states might be a complex of different states sharing the same kinetic characteristics. Nevertheless, experiments designed to estimate the amount of mRNA bound or not bound to miRISC and NOT1 can provide important information to validate this model.

## Summary and discussion

In this paper, we show that the current hypothesis about the sequence of interactions between miRISC, its target mRNA and the factor NOT1 is not supported by the data. We have shown that the mRNA is also degraded when the miRNA is not expressed, indicating the existence of an alternative pathway, possibly competing with the miRNA pathway.

We also show that when only miRNA is expressed (with NOT1 and PAN3 knocked down), the target mRNA is stabilized, probably because it is protected from the action of an alternative miRNA-independent pathway. We postulate that the binding between miRISC and mRNA is irreversible and leads to deadenylation independent decay of the target message in agreement with recent experimental studies. However, this assumption is not obligatory. Indeed, one could have hypothesized that binding to miRISC is reversible, and that the presence of miRNA alone just slows down the action of the alternative pathway. With the present data it is not possible to distinguish between these two alternatives.

Finally, our analysis indicates that the miRISC complex and NOT1 interact with each other *before *interacting with the mRNA. We assume that this discovery is not limited to the special miRNA-mRNA pair studied in [15] and is therefore a new general mechanism of mRNA control. Our analyses confirm the conclusions in [15] that PAN3 without NOT1 does not lead to an identifiable destabilization of the mRNA. Nevertheless, we see a strong cooperative effect between PAN3 and NOT1, where PAN3 is able to strongly enhance the binding of the miRISC+NOT1+PAN3 complex to the target mRNA compared to the miRISC+NOT1 complex alone.

Experimentally, one should be able to detect the presence of miRISC+NOT1 complexes in the absence of target mRNA in order to verify our findings. Moreover, steady state relative amounts of mRNA in the different biochemical states can provide further validation data for our networks and additional information to unveil further details of miRNA-mediated mRNA degradation.

## Competing interests

The authors declare that they have no competing interests.

## Authors' contributions

Conceived the project: AV, CS, DC; Data collection: CS; Model development: AV, CS, DC; Wrote the manuscript: AV, CS, DC.

## Supplementary Material

Additional file 1 **This file includes several explicit calculations, the numerical values of the data, and a summary of the theory used in this paper**. It includes also a table summarizing the values of the rates obtained upon fitting plus two additional fitting attempts with the originally proposed network to show that the fit does not perform very well. Click here for file

## Declarations

Publication of this article has been funded by the European Union FP7 project Marie Curie ITN "NICHE".

This article has been published as part of *BMC Systems Biology *Volume 9 Supplement 3, 2015: Proceedings of the Italian Society of Bioinformatics (BITS): Annual Meeting 2014: Systems Biology. The full contents of the supplement are available online at http://www.biomedcentral.com/bmcsystbiol/supplements/9/S3.
