# Supplementary material for: Single-molecule modeling of mRNA degradation by miRNA: Lessons from data
Source: BMC Syst Biol. 2015 Jun 1;9(Suppl 3):S2. doi: 10.1186/1752-0509-9-S3-S2 (PMC4464216; doi:10.1186/1752-0509-9-S3-S2)
Supplement: Additional file 1 — This file includes several explicit calculations, the numerical values of the data, and a summary of the theory used in this paper. It includes also a table summarizing the values of the rates obtained upon fitting plus two additional fitting attempts with the originally proposed network to show that the fit does not perform very well. [file 1752-0509-9-S3-S2-S1.pdf]

Supplementary Materials for
Single-Molecule Modeling of mRNA Degradation by miRNA: Lessons from Data

Celine Sin, Davide Chiarugi, Angelo Valleriani*
Department of Theory and Bio-Systems
Max Planck Institute of Colloids and Interfaces
14424 Potsdam, Germany

1 From single-molecule pathways to decay patterns

We model mRNA decay as a single molecule stochastic process. The process starts from an initial state 0, which represents the mRNA in its initial condition, and terminates in the degradation state n. The initial and the degradation states are defined here based on the time point from which the mRNA is experimentally detected until it is not detected anymore, respectively, in the context of the experimental technique used in [1]. Each realization of the stochastic process, which starts from state 0 to the degradation state through the network of states represents the life of a single mRNA molecule. Therefore, the distribution of the random time T elapsing from state 0 to the degradation state n can be identified with the distribution of the lifetimes of the mRNA molecules. The state space $\sigma = \{0, \ldots, n\}$ is thus made of n transient states and one absorbing state n (a generalization to more absorbing states is straightforward, see [2]).

In our modeling framework each state transition describes the occurrence of what we define a *first-order biochemical event*. This definition includes three possible biochemical scenarios:

(i) an elementary first-order chemical reaction

(ii) a pseudo first-order biochemical reaction: the actual reaction order is > 1 but the concentration all of the reactants except for one are at the steady state level. As a result, this reaction behaves as a “true” first-order reaction

(iii) an apparent first-order reaction: a complex chain of reactions with a single rate limiting step, thus exhibiting first-order behavior

In the context of the mRNA degradation pathway we consider the two latter cases only, due to the complexity of the involved biochemical reactions. If we assume that the

*email: angelo.valleriani@mpikg.mpg.de

probability of occurrence of each biochemical event depends only on the current state (Markov property), then we can model the stochastic processes describing the life of mRNA molecules as Markov chains.

Thus, following [2, 3, 4, 5], we can write the lifetime probability density ϕ as

$$\phi(t) \;=\; \vec{e}_0 \left[\exp\left(\mathbf{S}_0 t\right) S_A\right] \,, \tag{1}$$

where S_0 is a square $n \times n$ matrix. The off-diagonal elements of S_0 are the transition rates between the transient states, and the diagonal elements are composed of the negative sum over all outgoing transition rates from each transient state. The column vector S_A contains all the transition rates governing the transitions from the transient into the absorbing states. The row vector of length n $\vec{e}_0$ has a one in the position corresponding to the initial condition and zeros elsewhere.

As discussed in Ref. [5], we must take into consideration that at the beginning of the experiment, the expression of mRNA is at the steady state. Thus, not all mRNA molecules are found at the initial state 0; some of them will be in other states because they are older. If the transcription of mRNAs was ongoing long before the beginning of the decay experiment, one can assume that the age distribution of the population of mRNA is at steady state[1]. In [5] it was shown that when the age distribution is at steady state, the fraction of mRNAs remaining Δt time units after the stop of transcription is given by the function

$$\Lambda(\Delta t) \;=\; \frac{1}{\mathrm{E}(T)} \int_{\Delta t}^{\infty} \mathrm{d}t \; \left(1 - \Phi(t)\right) \,, \tag{2}$$

where $\mathrm{E}(T)$ is the average lifetime, namely the average value of T given by

$$\mathrm{E}(T) \;=\; \int_0^{\infty} \mathrm{d}t \; \left(1 - \Phi(t)\right) \,, \tag{3}$$

and Φ is the probability function of T given by

$$\Phi(t) \;=\; \int_0^{t} \mathrm{d}\tau \, \phi(\tau) \,, \tag{4}$$

where $\phi(t)$ can be computed from Eq. (1).

An important technical point for the computation of Λ is that in Eq. (2), $\mathrm{E}(T)$ is just the normalization factor in order to have $\Lambda(0) = 1$. The networks used in the main article are sufficiently simple, so that the calculation of ϕ can be done easily (see below), without resorting to the relatively complex formulation given in Eq. (1). Nevertheless, Eq. (1) is the general form of the function, which depends on the values of the rates in the matrix S_0. When Eq. (2) has been written in terms of the underlying rates, the rates can be determined by means of a nonlinear fitting of the log of $\Lambda(\Delta t)$ with the log of the data. The use of the logged data is justified by the common assumption that the experimental noise is multiplicative. Eqs. (1) and (2) include the case when there is just

[1]Short transcription pulses instead generate an age distribution away from steady state. This interesting extension of the theory will not be considered here because it is not relevant in this context.

one transient state, as a limiting case. This occurs when the decay pattern follows an exponential function whose parameter is the rate of degradation, and also the inverse of the average lifetime.

1.1 Fitting functions: General aspects

All our fits were performed for two or three parameters and compared with the exponential fit. Based on the Akaike Information Criterion (AIC) corrected for small sample sizes, we found that the fits with the exponential function did not perform sufficiently well to be considered in this study.

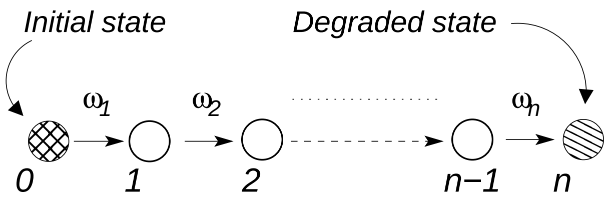

Figure 1: A generic chain made of n irreversible transitions. The probability density of the random time T from the initial to the degraded state is given by $\phi_n(t)$ derived in Eq. (5).

Since all our networks are comprised of irreversible transitions only, the computation of Λ can be split in the sub-tasks of computing the contribution of irreversible chains such as the one in figure 1. For each of these chains, the probability density ϕ_n for the absorption time in state n can be computed as the convolution of n independent exponential functions, and it is given as

$$\phi_n(t) \;=\; \left(\prod_{k=1}^{n} \omega_k\right) \cdot \left(\sum_{j=1}^{n} \frac{\exp(-\omega_j t)}{\prod_{k\neq j}(\omega_k - \omega_j)}\right) \,, \tag{5}$$

if all the ω_j are different from one another. In the networks considered in this work we just need the two cases corresponding to $n = 1$, *i.e.* the exponential function, and $n = 2$ because all other cases can be cast in form of a linear combination of these two cases. The cumulative functions associated to the ϕ_n are thus given by

$$\Phi_n(t) \;=\; 1 \;-\; \sum_{j=1}^{n} \left(\prod_{k\neq j} \frac{\omega_k}{\omega_k - \omega_j}\right) \exp(-\omega_j t) \,, \tag{6}$$

again under the assumption that all rates are different. As we have seen after fitting the data, this assumption is never violated.

Assume now that a given network has, say, three possible paths from the initial state to the absorbing state. Assume also that these paths are made of only irreversible transitions. Let us call these three paths a, b, and c and let us assume that these three

paths are characterized each by its own set of ω's and that the probability of each path to be taken is p_a, p_b and p_c, respectively. Let ϕ_a, ϕ_b and ϕ_c be the probability densities conditioned on each of the paths separately. Then, the total probability density ϕ is given by

$$\phi \;=\; p_a\phi_a + p_b\phi_b + p_c\phi_c \,, \tag{7}$$

and the cumulative probability function is given by

$$\Phi \;=\; p_a\Phi_a + p_b\Phi_b + p_c\Phi_c \,, \tag{8}$$

so the decay function Λ from Eq. (2) reads

$$\Lambda(\Delta t) \;\propto\; \int_{\Delta t}^{\infty} \mathrm{d}t \left(1 - \sum_x p_x \Phi_x(t)\right) \,, \tag{9}$$

where $x = a, b, c$, and the proportionality constant must be fixed so that $\Lambda(0) = 1$. If we now write the Φ_x as $\Phi_x + 1 - 1$ and we substitute, we obtain

$$\Lambda(\Delta t) \;\propto\; \sum_x p_x \mathcal{O}_x(\Delta t; \vec{\omega}_x) \,, \tag{10}$$

where we have defined

$$\mathcal{O}_x(\Delta t; \vec{\omega}_x) \;=\; \int_{\Delta t}^{\infty} \mathrm{d}t \; (1 - \Phi_x(t)) \;. \tag{11}$$

The formulation in Eq. (10) turns out to be a quite useful, since our Φ_n expressed in Eq. (6) take an explicit relatively simple form once put in Eq. (11). The vector $\vec{\omega}_x$ is the set of all ω's along the path x. Upon simple explicit integration we have thus, for $n = 1$ and $n = 2$ the two functions

$$\mathcal{O}_1(\Delta t; \omega_1) = \frac{\exp(-\omega_1 \Delta t)}{\omega_1} \,, \tag{12}$$

and

$$\mathcal{O}_2(\Delta t; \omega_1, \omega_2) = \frac{\omega_2 \exp(-\omega_1 \Delta t)}{\omega_1(\omega_2 - \omega_1)} + \frac{\omega_1 \exp(-\omega_2 \Delta t)}{\omega_2(\omega_1 - \omega_2)} \,, \tag{13}$$

to be used in the explicit calculations that follow.

1.2 Fitting functions: Specific forms

This section delivers the explicit form of the functions used in the fitting of the data.

- The generic two-state network in Figure 3 in the main article has

$$\begin{aligned} \Lambda(\Delta t) \;&\propto\; \frac{\mu}{\mu+\lambda} \mathcal{O}_1(\Delta t; \omega_1 = \lambda + \mu) \\ &+\; \frac{\lambda}{\mu+\lambda} \mathcal{O}_2(\Delta t; \omega_1 = \lambda + \mu, \omega_2 = \nu) \,, \end{aligned} \tag{14}$$

Δt	(-)	R	RP	RN	RNP
0	1	1	1	1	1
15	0.6899	0.6720	0.6722	0.5641	0.4885
30	0.5588	0.6238	0.6322	0.3206	0.2263
60	0.3375	0.4806	0.4184	0.1919	0.1272
180	0.1462	0.2262	0.2182	0.0766	0.0388
360	0.0857	0.1112	0.1035	0.0397	0.0217

Table 1: Experimental data as extracted from the plot published in [1]. The various experimental conditions are given in the first row: (-) = negative control, when the miRNA is not expressed; R = when only miRNA is expressed but both NOT1 and PAN3 are not expressed; RP = data when NOT1 is knocked down; RN = data when PAN3 is knocked down; RNP = all factors are present, no knock downs.

where in both cases $\omega_1 = \lambda + \mu$ takes into account that the dwell time on state A is exponential with parameter $\lambda + \mu$. This function was used to fit the data of the negative control (green line) and therefore to fix the rates λ, μ and ν. These three rates are kept fixed in all other networks.

- The networks in Figures 6, 8, 10 in the main article share the same structure and lead to

$$\begin{aligned} \Lambda(\Delta t) \quad &\propto \quad \frac{\lambda}{\lambda+\mu+\lambda'}\mathcal{O}_2(\Delta t; \omega_1 = \lambda+\mu+\lambda', \omega_2 = \nu) \\ &+ \quad \frac{\mu}{\lambda+\mu+\lambda'}\mathcal{O}_1(\Delta t; \omega_1 = \lambda+\mu+\lambda') \\ &+ \quad \frac{\lambda'}{\lambda+\mu+\lambda'}\mathcal{O}_2(\Delta t; \omega_1 = \lambda+\mu+\lambda', \omega_2 = \mu'), \end{aligned} \tag{15}$$

where $\lambda' = \lambda_R$, λ_{RN}, λ_{RNP} and $\mu' = \mu_R$, μ_{RN}, μ_{RNP} in Figures 6, 8, 10, respectively. To fit the data, as was done in Figures 7, 9, and 11 in the main article, we have kept the three rates λ, μ and ν fixed as they have been determined in the fit of the negative control. This means that the $\Lambda(\Delta t)$ given in Eq. (15) was used to fit the two additional rates λ' and μ' for each of the following three data sets.

1.3 Data used in the study

The experimental data used in this study have been extracted from [1]. We report the numerical values in table 1: the first column is the measurement time expressed in minutes; the second column is the data for the negative control, when the miRNA is not expressed, which was fitted with Eq. (14); the third column is the data when both NOT1 and PAN3 are knocked down (only miRNA is expressed) and was fitted with Eq.

rate	10^{-3}min^{-1}	10^{-3}min^{-1} 95% CI
λ	0.8	[0.2, 1.3]
μ	27.6	[22.9, 32.4]
ν	2.8	[1.8, 3.8]
λ_R	2.3	[0.0, 5.2]
μ_R	5.2	[3.0, 7.4]
λ_{RN}	46.0	[30.5, 68.7]
μ_{RN}	46.1	[31.9, 60.2]
λ_{RNP}	150.1	[90.8, 209.4]
μ_{RNP}	49.3	[37.3, 61.2]

Table 2: Values of the rates as they arise from the fitting of the experimental data. By using our derived functions Λ, Eqs. (14) or (15), we can perform a non-linear fit of the data listed in table 2. The values of the rates are given together with their 95% confidence interval (CI). Notice that μ_{RN} and μ_{RNP} are not significantly different whereas λ_{RN} and λ_{RNP} do differ significantly. This indicates that PAN3 enhances the recruitment of the target mRNA but not its degradation (see main text).

(15); the fourth column is the data when NOT1 is knocked down: since this data is very close to the data in the third column, an additional fit of it was not performed as these two sets of data cannot be distinguished from each other; the fifth column is the data when PAN3 is knocked down and was fitted with Eq. (15); the last column is the positive control, when all factors are expressed and was fitted with Eq. (15).

1.4 Fitting procedure and algorithm

Our fitting procedure consisted in finding the set of parameters $\vec{\omega}$ that minimized the square deviation of the logarithm of the data, as

$$\text{RSS} = \sum_{\{\Delta t\}} \left(\log(\text{data}(\Delta t)) - \log(\Lambda(\Delta t))\right)^2 , \tag{16}$$

where "data" is any of the columns in the table above and Λ is either Eq. (14) or Eq. (15) as explained above. The choice of the logarithm is dictated by the assumption that the noise is multiplicative. The minimization was done by using standard MATLAB routines ("lsqnonlin" and "nlinfit") leading to the same results. By using "nlinfit" we were able to estimate also the 95% confidence interval, reported together with the estimated values in the table 2:

Since the data concerns averages whereas the biochemical model for the degradation pathway is based on a single molecule perspective, it is to be expected that the confidence intervals are relatively large. In addition, the data used had to be extracted graphically from the plots published in [1] and thus are expected to contain random errors related to reading the data from the plots. Despite these shortcomings, any other of the alternative

condition	E[T] min	% via miRISC	$100\pi_0$	$100\pi_1$	$100\pi_2$
(-)	44.7	0.0	78.8	21.2	0
R	55.6	7.4	58.6	15.7	25.6
RP	52.7	5.5	63.1	16.9	19.9
RN	30.1	63.6	42.7	11.4	45.9
RNP	24.2	84.1	23.2	6.2	70.6

Table 3: Steady state properties. For each condition we compute the average lifetime of the mRNA (second column), the percent of molecules that are degraded via miRISC (third column) and the distribution of mRNA among the three main biochemical states of figure 2.

models we have tested were not able to fit the data (not shown), thus indicating that the networks proposed in this study are structurally correct.

1.5 Steady state properties and statistics

We can associate to each path of the type drawn in figure 1 its average absorption time given by

$$\mathrm{E}[T_n] = \sum_{j=1}^{n} \omega_j^{-1}, \tag{17}$$

which allows us to compute the average lifetime associated to each of the networks discussed in our paper. Therefore, the average lifetime associated to the decay pattern described by Eq. (14) is given by

$$\mathrm{E}[T^{(-)}] = \frac{\nu + \lambda}{\nu(\lambda + \mu)}, \tag{18}$$

where the upper index (-) indicates that this average lifetime refers only to the negative control decay data. Conversely, the average lifetime associated to the decay patterns described by Eq. (15) is given by

$$\mathrm{E}[T^{(RX)}] = \frac{\lambda\mu' + \lambda'\nu + \mu'\nu}{\mu'\nu(\lambda + \mu + \lambda')}, \tag{19}$$

where the upper index (RX) indicates that this refers to all analyzed networks where miRNA is involved together with none, one or both factors PAN3 and NOT1. When the rates are known one can compute the percent of target mRNA that are involved in miRNA mediated degradation, shown in the third column of table 3. Notice that the halftimes computed in [1] are very similar in value to our average lifetimes. The average lifetimes, however, have a clear meaning here because the steady state amount of mRNA is proportional to the average lifetime, whereas the halftimes only have a meaning in the framework of the exponential decay.

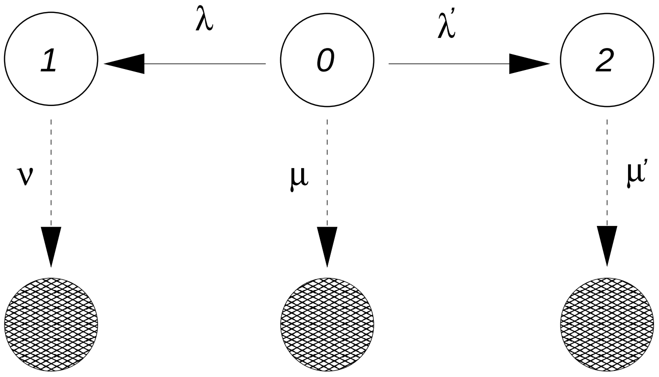

Figure 2: The network studied in this contribution is made of three states, 0, 1 and 2, whereas state 2 cannot be reached when $\lambda' = 0$ as in the case of the negative control. Using the rates and the Master equation we can compute the stationary distribution of the mRNA's in the three states for all models considered.

Another quantity of biological interest is the percent of mRNA in the different biochemical states of our network at steady state. The three states of the network are drawn in figure 2 and the stationary probabilities π_0, π_1 and π_2 can be computed from the Master equation by redirecting the arrows with rates μ, ν and μ' towards the initial state 0. This leads to the stationary distribution

$$\begin{aligned} \pi_0 &= \nu\mu'/(\nu\mu' + \lambda\mu' + \lambda'\nu) \\ \pi_1 &= \lambda\mu'/(\nu\mu' + \lambda\mu' + \lambda'\nu) \\ \pi_2 &= \nu\lambda'/(\nu\mu' + \lambda\mu' + \lambda'\nu) \end{aligned} \tag{20}$$

which have a clear limit when $\lambda' \to 0$ for the negative control case. The values of the probability distribution can be used to estimate how many mRNA should be found in the cell in the three different biochemical states, 0, 1 and 2 in figure 2. Notice that the % of mRNA degraded through miRNA targeting can also be computed in terms of normalized fluxes:

$$\% \text{ via miRISC} = \frac{\pi_2\mu'}{\pi_0\mu + \pi_1\nu + \pi_2\mu'}\,, \tag{21}$$

consistent with the values computed earlier, given in the third column of table 3.

2 Why the originally hypothesized pathway is not consistent with the experimental data

There are several reasons why the biochemical network proposed in [1] is not an adequate model for miRNA-mediated mRNA degradation:

1. The network proposed in [1] does not contain all the state transitions that are

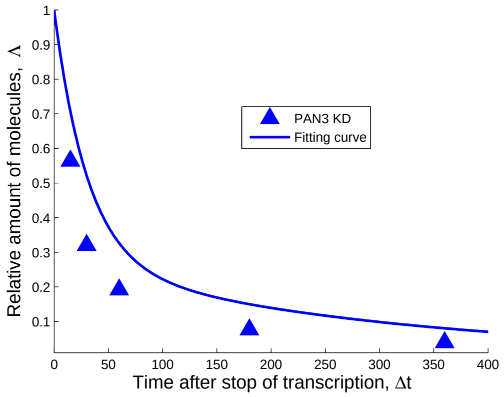

Figure 3: Fit of the data of the decay pattern when PAN3 has been knocked down. The model used here is the one of [1] complemented with a pathways for alternative routes to degradation. In our interpretation this means that $\lambda' = \lambda_R$ in figure 2 is fixed with the data of the experiment with both PAN3 and NOT1 knocked down, while also λ, μ and ν are kept fixed. The only parameter left free to be fixed with this set of data is therefore μ'. However, since too few mRNAs are targeted by miRISC alone, the extra parameter μ' is not sufficient to fit the data.

necessary for fitting both the negative control data and the data where the miRNA is knocked-down.

2. Through the action of miRISC alone, only about 7% of the target mRNA are degraded via the action of the miRNA (see second row, third column in table 3)

3. The hypothesis formulated by [1] foresees that NOT1 and PAN3 bind to the mRNA after the binding of miRISC. To mimic this scenario, we can thus impose $\lambda' = \lambda_R$ in figure 2, thereby implying that only one degree of freedom, namely the parameter μ', remains available for being tuned through the data fitting procedure. As seen in figures 3 and 4, the model proposed with this assumption does not provide a good fit to the data.

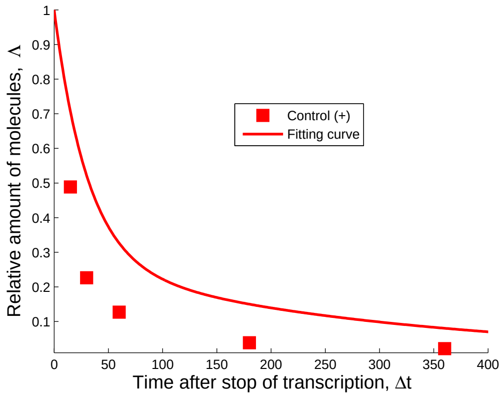

Figure 4: Fit of the data of the decay pattern when all factors are expressed (no knock downs). The model used here is the one proposed in [1] complemented with a pathway for alternative routes to degradation. In our interpretation this means that $\lambda' = \lambda_R$ in figure 2 is fixed with the data of the experiment with both PAN3 and NOT1 knocked down, while also λ, μ and ν are kept fixed. The only parameter left free to be fixed with this new set of data is therefore μ'. However, since too few mRNAs are targeted by miRISC alone, the extra parameter μ' turns out to be not sufficient to fit the data.

References

[1] Braun, J.E., Huntzinger, E., Fauser, M., Izaurralde, E.: GW182 proteins directly recruit cytoplasmic deadenylase complexes to miRNA targets. Molecular cell **44**(1), 120–133 (2011)

[2] Valleriani, A., Li, X., Kolomeisky, A.B.: Unveiling the hidden structure of complex stochastic biochemical networks. J Chem Phys **140**(6), 064101 (2014). doi:10.1063/1.4863997

[3] Valleriani, A., Liepelt, S., Lipowsky, R.: Dwell time distributions for kinesin's mechanical steps. EPL (Europhysics Letters) **82**(2), 28011–1280116 (2008)

[4] Keller, P., Valleriani, A.: Single-molecule stochastic times in a reversible bimolecular reaction. J Chem Phys **137**(8), 084106 (2012). doi:10.1063/1.4747337

[5] Deneke, C., Lipowsky, R., Valleriani, A.: Complex Degradation Processes Lead to Non-Exponential Decay Patterns and Age-Dependent Decay Rates of Messenger RNA. PLoS ONE **8**(2) (2013)
